# Repeated percutaneous hepatic perfusion with melphalan can maintain long-term response in patients with liver cancers

**DOI:** 10.1007/s00270-021-02983-2

**Published:** 2021-10-29

**Authors:** Rhea Veelken, Bettina Maiwald, Steffen Strocka, Tim-Ole Petersen, Michael Moche, Sebastian Ebel, Timm Denecke, Matus Rehak, Manuel Florian Struck, Dirk Forstmeyer, Sebastian Rademacher, Daniel Seehofer, Thomas Berg, Florian van Bömmel

**Affiliations:** 1 Division of Hepatology, Department of Medicine II, Leipzig University Medical Center, Liebigstr. 20, 04103 Leipzig, Germany; 2 Department of Diagnostic and Interventional Radiology, Leipzig University Medical Center, Liebigstr.20, 04103 Leipzig, Leipzig, Germany; 3 Clinic for Diagnostic and Interventional Radiology, Barbara Hospital Halle, St. Elisabeth and StMauerstr. 5, 06110 Halle (Saale), Germany; 4 Department of Interventional Radiology, Helios-Park-Klinikum Leipzig, Strümpellstraße 41, 04289 Leipzig, Germany; 5 Department of Ophthalmology, Leipzig University Medical Center, Liebigstr. 10-14, 04103 Leipzig, Germany; 6 Department of Anesthesiology and Intensive Care Medicine, Leipzig University Medical Center, Liebigstr. 20, 04103 Leipzig, Germany; 7 Division of Oncology, Department of Medicine II, Leipzig University Medical Center, Liebigstr. 22, 04103 Leipzig, Germany; 8 Department of Visceral, Thoracic and Vascular Surgery, Leipzig University Medical Center, Liebigstr. 20, 04103 Transplant, Germany; 9 University Liver Tumor Center (ULTC), Leipzig University Medical Center, Liebigstr. 22, 04103 Leipzig, Germany

**Keywords:** Liver cancer, Uveal melanoma, Chemosaturation, Melphalan

## Abstract

Chemosaturation (CS; CHEMOSAT®, Delcath Systems Inc.) temporarily administers melphalan into the liver by percutaneous hepatic perfusion (PHP). CS-PHP can effectively control growth in liver tumors, but efficacy and tolerability of sequential treatments are unclear. We analyzed outcomes of sequential CS-PHP treatment. Patients with either unresectable intrahepatic metastases of ocular melanoma (OM, *n* = 9), cholangiocarcinoma (CCA, *n* = 3), or hepatocellular carcinoma (HCC, *n* = 1) were recruited retrospectively. Response was assessed by tomography imaging. Ten patients (mean age 60 years) with more than one CS-PHP treatment were included. CS-PHP was administered 2–6 times in the OM patients, 3 times in the CCA, and the HCC patient received 6 treatments. Overall response rate (ORR) to CS-PHP was 80%, and stable disease was achieved in one patient. Median hepatic progression-free survival (hPFS) was 336 days (range 0–354) for OM, 251 days for the CCA patient, and 256 days for the HCC patient. At the end of observation (153–701 days after first CS-PHP), 6/10 patients were still alive (5/9 with OM, 0 with CCA, and 1 with HCC). Death cases were not related to CS-PHP. Adverse events were mostly hematologic, grade I-IV, and self-resolving. The liver function was not deteriorated by CS-PHP. We conclude that repeated CS-PHP treatments were effective and well tolerated in the long term.

**Supplementary Information:**

The online version contains supplementary material available at 10.1007/s00270-021-02983-2.

Chemosaturation or percutaneous hepatic perfusion (CS-PHP) with melphalan represents a regional therapy strategy for unresectable primary or secondary intrahepatic malignancies. A randomized controlled phase III trial assessed the efficacy of CS-PHP in patients with liver metastases of cutaneous or ocular melanoma (OM) [1]. Treatment with CS-PHP was associated with superior prolonged median hepatic progression-free survival (hPFS; 7 vs. 1.6 months, respectively) and improved hepatic objective response compared to best alternative care. These encouraging results are supported by other small, non-randomized studies in OM patients [1–3]. CS-PHP also showed response in some patients with hepatic metastases from neuro-endocrine tumors, sarcomas, and various types of carcinomas [1–10]. Moreover, the safety of CS-PHP and promising outcomes in a patient population with primary or secondary tumors were recently demonstrated in a single-center study [11]. Although CS-PHP treatment seems to carry a significant benefit for some patients, the optimal frequency, safety, and the tolerability of CS-PHP treatment repetition are undefined. Thus, most patients reported in the present studies received in general a minimum of two cycles of CS-PHP [6, 12]. We are interested in the question of whether CS-PHP planned from the outset as a repeat treatment is a more effective approach to the long-term treatment of suitable patients. Therefore, based on the decision of the tumor board, sequential treatments were performed in eligible patients at our center.

Accordingly, between 01/2016 and 12/2019, 13 patients with OM, CCA, or hepatocellular carcinoma (HCC) were treated with CS-PHP at our center, and 10 of them received multiple treatments, with a minimum of two CS-PHP procedures and on which we want to focus in this work. Prerequisites for CS-PHP treatment were sufficient hematologic, renal, and hepatic function and consent of the patient, as well as an Eastern Cooperative Oncology Group (ECOG) 0–1. Significant arterial hepatico-enteric anastomoses were embolized to prevent systemic exposure to melphalan during angiographic evaluation up to 14 days prior to CS-PHP. Before administration of melphalan, a venogram was obtained through the injection port of the double-balloon catheter to exclude leakages [13]. Patients received CS-PHP under general anesthesia and with systemic anticoagulation using the Hepatic CHEMOSAT® Delivery System (Delcath Systems, Inc., NY, the USA) according to the manufacturer’s recommendations [7].

In total, 45 CS-PHPs were performed in 13 patients with a maximum of 6 treatments per patient. Nine patients had unresectable intrahepatic metastases of OM, two of which also had tumor pulmonary tumor manifestations, peritoneal carcinomatosis (*n* = 1), osseous (*n* = 1), subcutaneous (*n* = 2), or cerebral metastasis (*n* = 1)**.** Two patients with CCA received the initial CS-PHP treatment and one another three subsequent treatments. The HCC patient received 6 CS-PHP treatments as an individual case decision, because of intolerance to tyrosine kinase inhibitors and that immune checkpoint therapy was not sufficiently established at the time of the treatment interval. Three patients with only one CS-PHP treatment were excluded for the analysis. Patient 3 and 13 showed a fulminant hepatic tumor progression after the first CS-PHP and died due to too rapid tumor progression. Patient 11 suffered from severe symptoms of liver cirrhosis and died because of esophageal varices bleeding due to portal hypertension caused by portal vein thrombosis (Fig. 1).

**Fig. 1:**
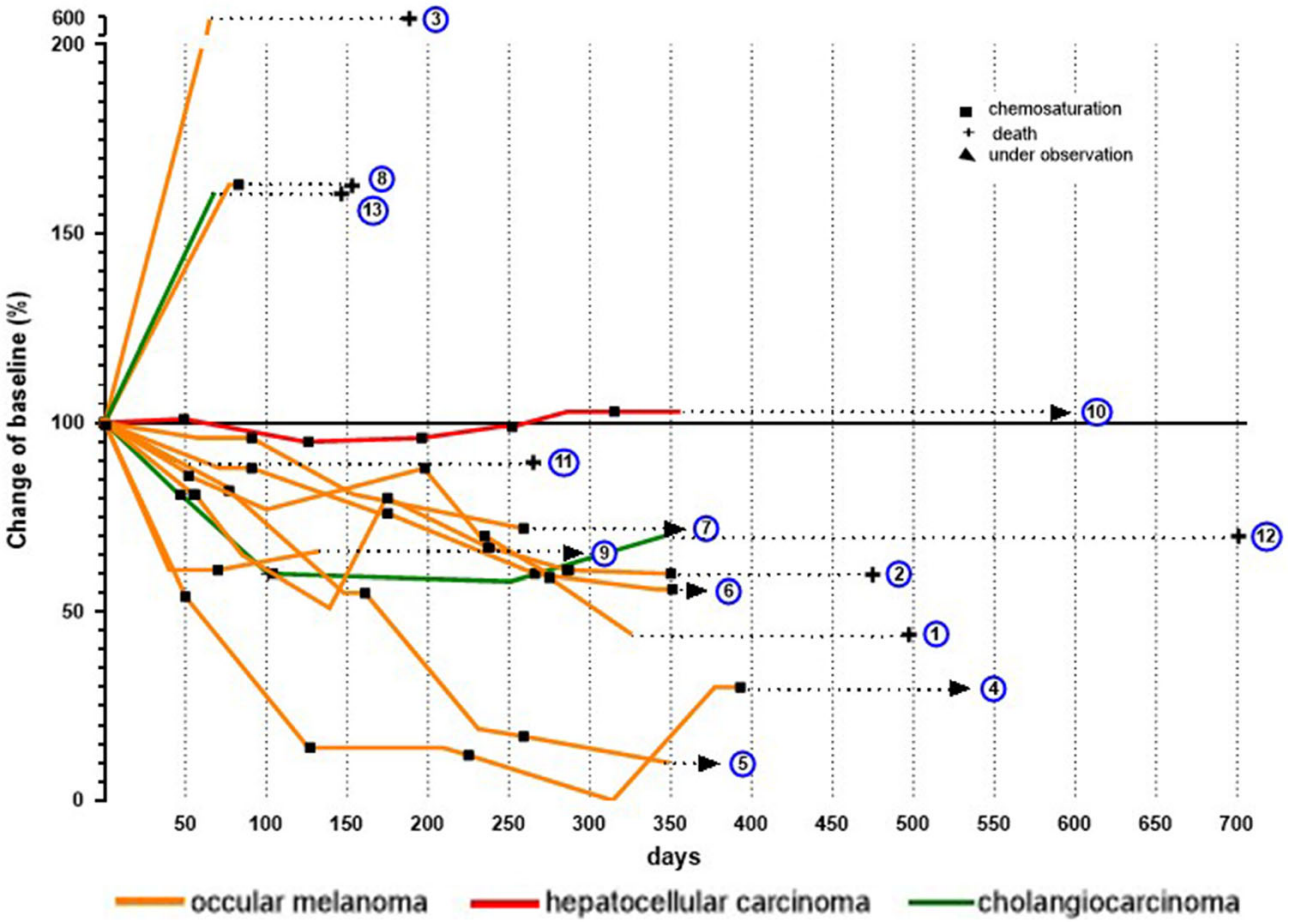
Individual changes of tumor sizes following CS-PHP treatment

At the time of data collection, six out of ten patients were in medical follow-up for a median duration of 361 (range, 284–585) days from first CS-PHP and 1602 (range, 643–2104) days from first diagnosis of primary malignant disease. A total of four patients died within the observation period. Three patients died despite intrahepatic tumor response from cardio-vascular events (patient 1), cerebral OM metastasis (patient 2), or liver function loss following a transarterial radio-embolization, which was conducted due to the patient’s preference about one year since the last CS-PHP treatment (patient 12). Patient 8 died early after the second CS-PHP treatment due to rapid tumor progression. No cases of deaths were directly linked to CS-PHP treatment.

The ORR to CS-PHP according to tumor decrease by mRECIST criteria was 80%, including seven patients with OM and the one with CCA assessed at first re-staging date 8–10 weeks after treatment. Stable intrahepatic disease was achieved in one patient with HCC (patient 10) over a duration of 20 months.

The median hPFS for OM patients was 336 (range, 0–354) days. The hepatic tumor burden in the single HCC patient was stable during the entire observation period of 356 days, and no extrahepatic progress was detected. The hPFS for the CCA patient was 251 days.

The median OS of the overall population was 421 (range, 153–701) days from first CS-PHP. The OM patients showed a median OS of 391 (range, 153–523) days from first CS-PHP.

CS-PHP induced massive tumor shrinkage in individual patients, as demonstrated in a 30-year-old female (patient 2) with multiple hepatic OM metastases with a diameter of up to 10 cm. The tumor burden showed a strong decrease of 50% and 43% after the first and second CS-PHP treatment, respectively, and the tumor extension remained stable over 13 months (Fig. 2A). Another OM patient showed stable disease over 6 CS-PHP treatments (patient 10). In another patient (patient 4) with multiple hepatic OM metastases, complete response was found after 4 CS-PHP treatments (Fig. 2B). Finally, only one OM patient who initially showed response to CS-PHP had intrahepatic tumor progression after the fourth CS-PHP treatment, 7 months after first CS-PHP.

**Fig. 2:**
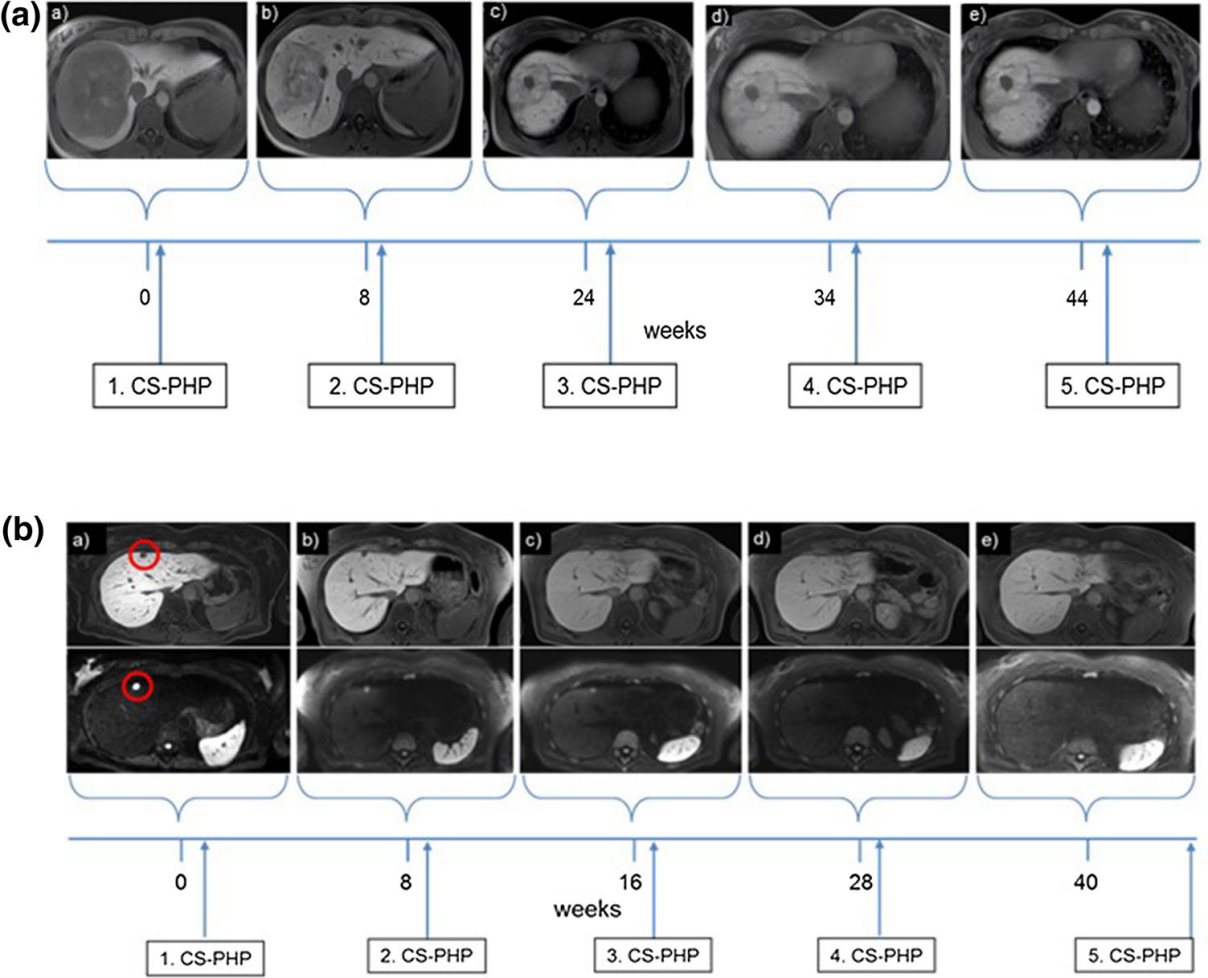
**A** Example for response of multiple (> 10) liver metastasis of uveal melanoma by repeated CS-PHP treatment (patient 2). At the start of CS-PHP treatment, a large liver metastasis in segment 7/8 (10 cm) as well as smaller herds in both liver lobes (up to 2.1 cm) was present **(a)**. Following the first cycle, the tumor burden decreased by 50% **(b)**, and by another 43% after the second cycle **(c)**. Intrahepatic metastases remained stable during following CS-PHP treatments **(d,e)**. The patient died from rapidly progressive brain metastases 17 months after initiation of CS-PHP. **B** Example for local control of multiple (> 10) liver metastasis of uveal melanoma by repeated CS-PHP treatment (patient 4). Magnetic resonance imaging with intravenous contrast depicts a target lesion (red cycle) in segment IVa (12 mm) before first CS-PHP **(a)**. The target lesion remained stable after the initial CS-PHP (12 mm) **(b)**. After the second CS-PHP, the target lesion shows a stable diameter of 10 mm **(c)**. After the third and fourth CS-PHP, the target lesion shows partial (8 mm) and finally complete response, respectively **(d)** and **(e)**. Top row: T1 post-contrast (hepatobiliary phase). Bottom row: diffusion weighted imaging *(b 1000)*

Adverse events associated with CS-PHP were classified according to the Common Terminology Criteria for adverse events (CTCAEv4.03). A total of 96 adverse events (AEs) were reported during the CS-PHP treatments, including 52 classified grade I, 25 grade II, 16 grade III, and three grade IV (Supplement 1). All AEs occurred within 15 days after CS-PHP treatment were transient and, with exception of one case, self-resolving. Thus, no patient suffered a severe side effect directly linked to melphalan application. The majority of AEs were hematologic. In one case (patient 10), neutropenic fever (> 38.5 °C) occurred after the first CS-PHP treatment which was treated with filgrastim and erythropoietin for bone marrow stimulation. During the subsequent 5 CS-PHP treatment, no additional neutropenia occurred. One patient with OM (patient 9) suffered circulatory instability, cardiac and ventilator insufficiency during melphalan administration. However, it is important to point out that a statement about the tolerability in patients with liver cirrhosis cannot be made conclusively.

Over the course of the repeated CS-PHP treatments, a moderate increase in median alanine aminotransferase (ALT) was observed. Median levels of bilirubin remained unchanged, and only some patients showed mild bilirubin increases during CS-PHP repetition (Fig. 3a-b).

**Fig. 3:**
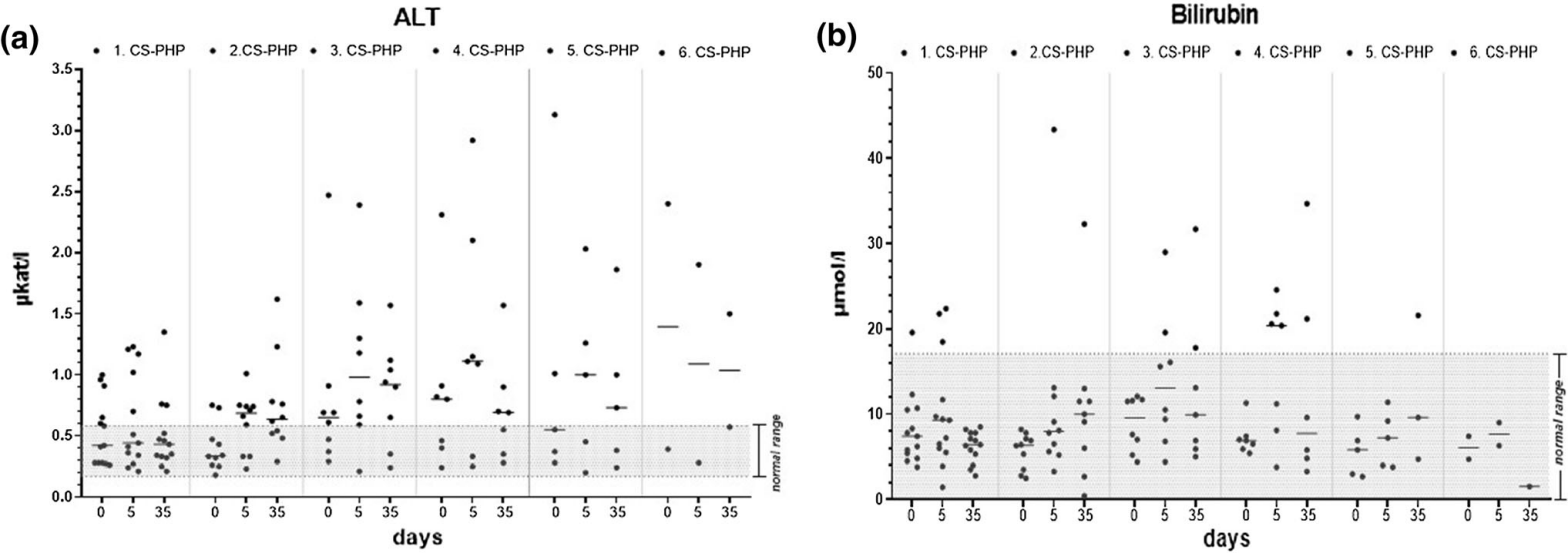
Individual levels and medians of ALT **(a)** and bilirubin **(b)** of the 13 patients at days 0, 5 and 35 after each CS-PHP treatment

Thus, most patients reported in the present studies received in general two cycles of CS-PHP, with the exception of a small group of patients with more than two repetitive treatments [7, 12–16].The analysis of our long-term approach showed that up to 6 CS-PHP can be well tolerated, in alignment with results of a recently published single-center analysis [11]. Even after multiple repetitions, there was no increase in CS-PHP-related AEs. It therefore seems justified to assume that even more repetitions of CS-PHP treatments as applied in our population would be tolerable in individual patients. Importantly, patients who had initially responded to treatment showed stable disease or further intrahepatic response during subsequent CS-PHP treatments.

Another argument for long-term repetition of CS-PHP deduced from our findings is that the initial decrease in tumor mass seen in all patients showing an initial response to CS-PHP remained stable during the observation period, and continued to further decrease after treatment repetitions, even under the visualization limit.

In summary, our findings are encouraging to study the repetitive long-term use of CS-PHP treatment as a novel therapeutic approach for hepatic OM metastasis. Moreover, also, patients with primary liver tumors might benefit from this treatment. Further studies are warranted to develop CS-PHP as an effective treatment option for primary and secondary liver tumors.

## Supplementary Information

Below is the link to the electronic supplementary material.

Supplementary file1 (DOCX 18 KB)

## Abbreviations

CS: Chemosaturation
PHP: Percutaneous hepatic perfusion
ACT: Activated clotting time
ORR: Overall response rate
OS: Overall survival
hPFS: Hepatic progression-free survival
mRECIST: Modified Response Evaluation Criteria In Solid Tumors
CTCAEv4.03: Common Terminology Criteria for adverse events
OM: Ocular melanoma
CCA: Cholangiocarcinoma
HCC: Hepatocellularcarcinoma
ULN: Upper limit of normal
APTT: Activated Partial Thromboplastin Time
PT: Prothrombin time
AST: Aspartate aminotransferase
ALT: Alanine aminotransferase
ALP: Alkaline phosphatase
LDH: Lactic acid dehydrogenase
SI: International System of Units
ECOG: Eastern Cooperative Oncology Group
