# Supplementary material for: Repeated percutaneous hepatic perfusion with melphalan can maintain long-term response in patients with liver cancers
Source: Cardiovasc Intervent Radiol. 2021 Oct 29;45(2):218–22. doi: 10.1007/s00270-021-02983-2 (PMC8555734; doi:10.1007/s00270-021-02983-2)
Supplement: Supplementary file 1 — Supplementary file1 (DOCX 18 KB) [file 270_2021_2983_MOESM1_ESM.docx]

| **Supplement 1:** Adverse events according to CTCAE classification occurring after CS-PHP treatment | | | | | | | | | | | | | | | | | | | |  |
| --- | --- | --- | --- | --- | --- | --- | --- | --- | --- | --- | --- | --- | --- | --- | --- | --- | --- | --- | --- | --- |
| **Underlying disease** | **Patient no.** | **1. CS-PHP** | | **2. CS-PHP** | | **3. CS-PHP** | | | **4. CS-PHP** | | | **5. CS-PHP** | | | **6. CS-PHP** | | | | |  |
|  |  | AE | Grade | AE | Grade | AE | | Grade | AE | | Grade | AE | | Grade | AE | | | Grade | |  |
| **OM** | 1 | Anemia | I | Anemia | I | Anemia | | II | Anemia | | I | Anemia | | II |  | | | | |  |
|  |  |  |  | Leuko  penia | I |  |  |  |  |  |  |  |  |  |  |  |  |  |  |  |
|  |  | Thrombopenia | III | Thrombopenia | I | Thrombopenia | | IV | Leuko  penia | | II | Leuko  penia | | III |  |  |  |  |  |  |
|  |  |  |  |  |  |  |  |  | Thrombopenia | | I | Thrombopenia | | IV |  |  |  |  |  |  |
|  | 2 | Anemia | I | Anemia | II | Anemia | | II | Anemia | | II | Anemia | | I | Anemia | | | I | |  |
|  |  |  |  |  |  |  |  |  |  |  |  |  |  |  | Leuko  penia | | | II | |  |
|  |  |  |  | Thrombopenia | I | Nausea | | I | Thrombopenia | | I | Thrombopenia | | I | Thrombopenia | | | II | |  |
|  | 3 | Anemia | I |  | |  | | |  | | |  |  |  |  |  |  |  |  |  |
|  |  | Thrombopenia | I |  |  |  |  |  |  |  |  |  |  |  |  |  |  |  |  |  |
|  | 4 | Anemia | I | Anemia | II | Anemia | | III | Anemia | III | | Anemia | | I |  |  |  |  |  |  |
|  |  | Thrombopenia | I | Thrombopenia | II | Thrombopenia | | III | Thrombopenia | III | | Thrombopenia | | I |  |  |  |  |  |  |
|  | 5 | Anemia | I | Anemia | I | Anemia | | II | Anemia | I | |  | | | | |  |  |  |  |
|  |  | Thrombopenia | I | Thrombopenia | I | Thrombopenia | | I | Thrombopenia | I | |  |  |  |  |  |  |  |  |  |
|  | 6 | none | | Anemia | I | Anemia | | II | Anemia | II | | Anemia | | I |  |  |  |  |  |  |
|  |  |  |  | Thrombopenia | I | Thrombopenia | | I | Thrombopenia | I | | Thrombopenia | | III |  |  |  |  |  |  |
|  | 7 | Anemia | I | Anemia | I | Anemia | | I | Anemia | I | |  | | |  | | | | | |
|  |  | Thrombopenia | I | Thrombopenia | I | Thrombopenia | | II | Thrombopenia | I | |  |  |  |  |  |  |  |  |  |
|  | 8 | Anemia | I | Anemia | III |  | | |  | | |  | | |  | | | | | |
|  |  | Thrombopenia | I | Thrombopenia | I |  |  |  |  |  |  |  |  |  |  |  |  |  |  |  |
|  | 9 | Thrombopenia | I | Anemia | I |  | | | | | |  |  |  |  |  |  |  |  |  |
|  |  |  |  | Thrombopenia | III |  |  |  |  |  |  |  |  |  |  |  |  |  |  |  |
| **HCC** | 10 | Anemia | III | Anemia | II | Anemia | | I | Anemia | II | | Anemia | | I | Anemia | | | II | |  |
|  |  | Neutro  penia | III | Leuko  penia | II | Leuko  penia | | II | Leuko  penia | III | | Leuko  penia | | III | Fatigue | | | I | |  |
|  |  | Neutro  penic fever | III |  |  |  |  |  |  |  |  |  |  |  | Nausea | | | I | |  |
|  |  |  |  | Thrombopenia | I | Thrombopenia | | II | Thrombopenia | II | | Thrombopenia | | II | Thrombopenia | | | I | |  |
| **CCA** | 11 | Thrombopenia | II |  | |  | | |  | | |  | | |  | | | | |  |
|  | 12 | Anemia | I | Thrombopenia | III | Fatigue | | I |  | | |  | | |  | | | | |  |
|  |  | Thrombopenia | III |  |  | Thrombopenia | | II |  |  |  |  |  |  |  |  |  |  |  |  |
|  | 13 | Fatigue | II |  | | |  | |  | | | |  | | |  | | |  |  |
|  |  | Leuko  penia | IV |  |  |  |  |  |  |  |  |  |  |  |  |  |  |  |  |  |
|  |  | Thrombopenia | I |  |  |  |  |  |  |  |  |  |  |  |  |  |  |  |  |  |
| CTCAE, Common Terminology Criteria for Adverse Events; CS-PHP, chemo saturation- percutaneous hepatic perfusion; AE, adverse event; OM, ocular melanoma; HCC, hepatocellular carcinoma; CCA, cholangio carcinoma. | | | | | | | | | | | | | | | | | | |  |  |
|  | | | | | | | | | | | | | | | | | | |  |  |
